# 969. Outbreak of Fungal Meningitis in US Patients who Received Surgical Procedures under Epidural Anesthesia in Matamoros, Mexico

**DOI:** 10.1093/ofid/ofad500.2463

**Published:** 2023-11-27

**Authors:** Dallas J Smith, Samantha Williams, Anastasia P Litvintseva, Nirma D Bustamante, Luis Ostrosky-Zeichner, Simone Godwin, Jessica C Pearson, JulieAnna Rivas, Nicole Evert, Thi Dang, Angel Guevara, Lori Koenecke, Tom M Chiller, Jeremy A W Gold

**Affiliations:** Mycotic Diseases Branch, Centers for Disease Control and Prevention, Atlanta, GA; Centers for Disease Control and Prevention, Atlanta, Georgia; Centers for Disease Control and Prevention, Atlanta, Georgia; CDC, El Paso, Texas; McGovern Medical School. UTHealth, Texas, Texas; Tennessee Department of Health, Nashville, Tennessee; Missouri Department of Health and Senior Services, Raytown, Missouri; Arizona Department of Health Services, Phoenix, Arizona; Texas Department of State Health Services, Austin, Texas; Texas Department of State Health Services, Austin, Texas; Texas Department of State Health Services, Austin, Texas; South Dakota Department of Health, Pierre, South Dakota; Centers for Disease Control and Prevention, Atlanta, Georgia; Centers for Disease Control and Prevention, Atlanta, Georgia

## Abstract

**Background:**

US public health officials are responding to a multinational, multistate fungal meningitis outbreak involving travelers who had primarily cosmetic procedures (e.g., liposuction) under epidural anesthesia at 2 Matamoros, Mexico clinics (closed 5/13/2023 by Mexican authorities). *Fusarium solani* species complex fungal DNA was identified in cerebral spinal fluid (CSF) by polymerase chain reaction and next-generation metagenomic sequencing; a highly resistant strain was isolated from a single tissue culture. We describe epidemiology, demographics, and clinical features to inform prevention messaging, guidance, and future responses.

**Methods:**

Public health officials collected data on confirmed and probable cases (1/1/23–7/25/2023) using a standardized case report form; data were analyzed in Microsoft Excel.

**Results:**

In total, 10 probable (1 death) and 10 confirmed (8 deaths) US fungal meningitis cases were identified; all involved epidural anesthesia from the same anesthesiologist at both clinics. Most cases (80%) occurred in Texas residents. Median patient age was 31 years (range: 23–52); 19 patients were women; 13 were Hispanic/Latino. None had underlying conditions reported. At least 4 patients were uninsured, with several reporting associated care-seeking delays. Common symptoms were headache (n=18), nausea (n=13), fever (n=12), and stiff neck (n=12). Median (IQR) initial CSF results were white blood cell count (498/µL [343–825]), glucose (31 mg/dL [26–40]), and protein (99 mg/dL [52–2140]). In 8 patients, CSF (1,3)-β-D-Glucan was ≥ 500 pg/ml. From procedure date, average days (range) to symptom onset was 19 (0–58), to hospitalization was 52 (14–106).

**Conclusion:**

In the US, the fungal meningitis outbreak has primarily affected young, healthy Hispanic/Latino women travelling to Matamoros, Mexico, for procedures and resulted in a high mortality rate (40%). CDC advises US residents against having elective procedures in Matamoros involving epidural anesthetic injection. Insurance barriers may have led to medical tourism to Mexico and subsequent delayed treatment for fungal meningitis, highlighting the need for preparation to address healthcare access disparities in future outbreaks.

**Disclosures:**

**Luis Ostrosky-Zeichner, MD, FACP, FIDSA, FSHEA, FECMM, CMQ**, Astellas: Advisor/Consultant|Astellas: Grant/Research Support|Astellas: Honoraria|F2G: Advisor/Consultant|F2G: Grant/Research Support|F2G: Honoraria|Gilead: Advisor/Consultant|Gilead: Grant/Research Support|Gilead: Honoraria|GSK: Advisor/Consultant|GSK: Grant/Research Support|GSK: Honoraria|Melinta: Advisor/Consultant|Melinta: Grant/Research Support|Melinta: Honoraria|NIH: Grant/Research Support|Pfizer: Advisor/Consultant|Pfizer: Expert Testimony|Pfizer: Honoraria|Pulmocide: Advisor/Consultant|Pulmocide: Grant/Research Support|Pulmocide: Honoraria|Scynexis: Advisor/Consultant|Scynexis: Grant/Research Support|Scynexis: Honoraria|T2 biosystems: Advisor/Consultant|T2 biosystems: Grant/Research Support|T2 biosystems: Honoraria|Viracor: Advisor/Consultant|Viracor: Grant/Research Support|Viracor: Honoraria

